# Towards sustainable fishery management for skates in South America: The genetic population structure of *Zearaja chilensis* and *Dipturus trachyderma* (Chondrichthyes, Rajiformes) in the south-east Pacific Ocean

**DOI:** 10.1371/journal.pone.0172255

**Published:** 2017-02-16

**Authors:** Carolina Vargas-Caro, Carlos Bustamante, Michael B. Bennett, Jennifer R. Ovenden

**Affiliations:** 1 Shark and Ray Research Group, School of Biomedical Sciences, The University of Queensland, Brisbane, St Lucia, Queensland, Australia; 2 Molecular Fisheries Laboratory, School of Biomedical Sciences, The University of Queensland, Brisbane, St Lucia, Queensland, Australia; National Cheng Kung University, TAIWAN

## Abstract

The longnose skates (*Zearaja chilensis* and *Dipturus trachyderma)* are the main component of the elasmobranch fisheries in the south-east Pacific Ocean. Both species are considered to be a single stock by the fishery management in Chile however, little is known about the level of demographic connectivity within the fishery. In this study, we used a genetic variation (560 bp of the control region of the mitochondrial genome and ten microsatellite loci) to explore population connectivity at five locations along the Chilean coast. Analysis of *Z*. *chilensis* populations revealed significant genetic structure among off-shore locations (San Antonio, Valdivia), two locations in the Chiloé Interior Sea (Puerto Montt and Aysén) and Punta Arenas in southern Chile. For example, mtDNA haplotype diversity was similar across off-shore locations and Punta Arenas (*h* = 0.46–0.50), it was significantly different to those in the Chiloé Interior Sea (*h* = 0.08). These results raise concerns about the long-term survival of the species within the interior sea, as population resilience will rely almost exclusively on self-recruitment. In contrast, little evidence of genetic structure was found for *D*. *trachyderma*. Our results provide evidence for three management units for *Z*. *chilensis*, and we recommend that separate management arrangements are required for each of these units. However, there is no evidence to discriminate the extant population of *Dipturus trachyderma* as separate management units. The lack of genetic population subdivision for *D*. *trachyderma* appears to correspond with their higher dispersal ability and more offshore habitat preference.

## Introduction

The skates (Family Rajidae) form one of the largest groups within the batoids, with 27 genera and about 250 species [1, 2]. Among these, longnose skates (Tribe Rajini) have a worldwide distribution, inhabiting mostly marine environments from the sublittoral zone to depths of about 3,000 m [1, 2]. Life history traits such as slow growth, late sexual maturity, and low fecundity make them highly susceptible to overexploitation [3–7]. Appropriate management strategies require an understanding of the population structure of target species, especially as fishing pressure varies among species and fisheries [7]. Vulnerability to collapse due to direct and indirect fishing effects has been well documented for several species, including the common skate *Dipturus batis* (L. 1758), smooth skate *D*. *innominatus* (Garrick & Paul 1974), barndoor skate *D*. *laevis* (Mitchill 1818), longnosed skate *D*. *oxyrinchus* (L. 1758), white skate *Rostroraja alba* (Lacepède 1803), and the thornback ray *Raja clavata* L. 1758; where local extinctions have occurred as a result of continuous, unregulated fisheries [6–12].

Most studies on skates rely on fishery catch data to define a species’ distribution, abundance, dispersal potential and to detect demographic fluctuations [13, 14]. However, such data are generally insufficient to allow different stocks to be distinguished, as is required for sound fisheries management practices [15]. Molecular analysis has become an important tool in the study of exploited fish populations [16], and is frequently applied to examine stock structure and connectivity, and to identify species from body parts, such as fins and carcasses [17–23], each of which can aid fisheries management and conservation efforts [22–25].

The yellownose skate *Zearaja chilensis* (Guichenot 1848) and the roughskin skate *Dipturus trachyderma* (Krefft & Stehmann 1975) are an important component of commercial elasmobranch fisheries in South American waters [26–30]. Both of these longnose skates have a southern distribution from central Chile (32°S) to central Argentina (40°S), and includes waters off the Falkland Islands, although the Atlantic range of *Z*. *chilensis* extends northwards to the La Plata River (34°S) [31, 32]. The external morphology of these two skates is remarkably similar, especially in early life stages. As a result, frequent confusion of the species has occurred in landing data and official statistical records, which has impacted the effectiveness of fishery monitoring [28, 33– 34].

In Chile, a directed fishery for both species started in 1979, which initially targeted *Z*. *chilensis* [35]. However, until 2004 at least six species, including *Z*. *chilensis* and *D*. *trachyderma*, were landed under the generic category of ‘skate’ according to official records [36–38]. The fishing effort increased after 1993 when the fishery was opened to the Asian market. The artisanal fleet expanded due to international investment and, together with the industrial fishery, reported 3,000 tonnes (t) in landings in 1994. Once the Asian financial crisis ended in 1998, skate landings increased considerably, peaking 4,000 t and 5,193 t in 2000 and 2003 respectively [15, 38]. After 2006, a temporary fishing closure was imposed on the artisanal fleet, which operates during the austral summer months (December—February) to protect ‘possible reproductive events’ [33, 34]. Subsequently, between 2009 and 2011, a total fishing closure was imposed on the entire fishery in response to a declining trend in the overall catch per unit effort (CPUE), including the decline in the size of individuals landed. In spite of these ‘closures’, the government continues to allocate national catch quotas of up to 700 t annually [15, 28].

The longnose skate fishery in Chile is geographically extensive (approximately 20° of latitude, or 2,400 km from north to south), and operates year-round along the outer continental shelf at 150–450 m depth using bottom-set longlines [15, 28]. However, most of the fishing effort is located between central-south Chile, between 33.5°S and 45.5°S [28, 33, 34]. The fishery captures mostly small, immature fishes which, as a gauntlet fishery, could potentially compromise the integrity of the stock [28, 33]. The overall abundance of skates has declined substantially over the last decade due to intensive fishing pressure, and as consequence, the fishery is considered to be ‘fully exploited’ [13, 15, 28].

Considering the external morphological similarities between longnose skates, the current and past fishing pressures, and the overlap in both depth and latitudinal distribution; the aim of the present study was to assess genetic diversity and population structures of *Z*. *chilensis* and *D*. *trachyderma* in Chilean waters using mitochondrial and nuclear genetic markers. Population structure of each species was expected to be similar due to low fecundity and low dispersal potential. The research was carried out to provide relevant information to the single stock harvesting strategy for both species, currently used by the Chilean Government.

## Materials and methods

### Sample collection, species identification, and DNA extraction

**Fig 1:**
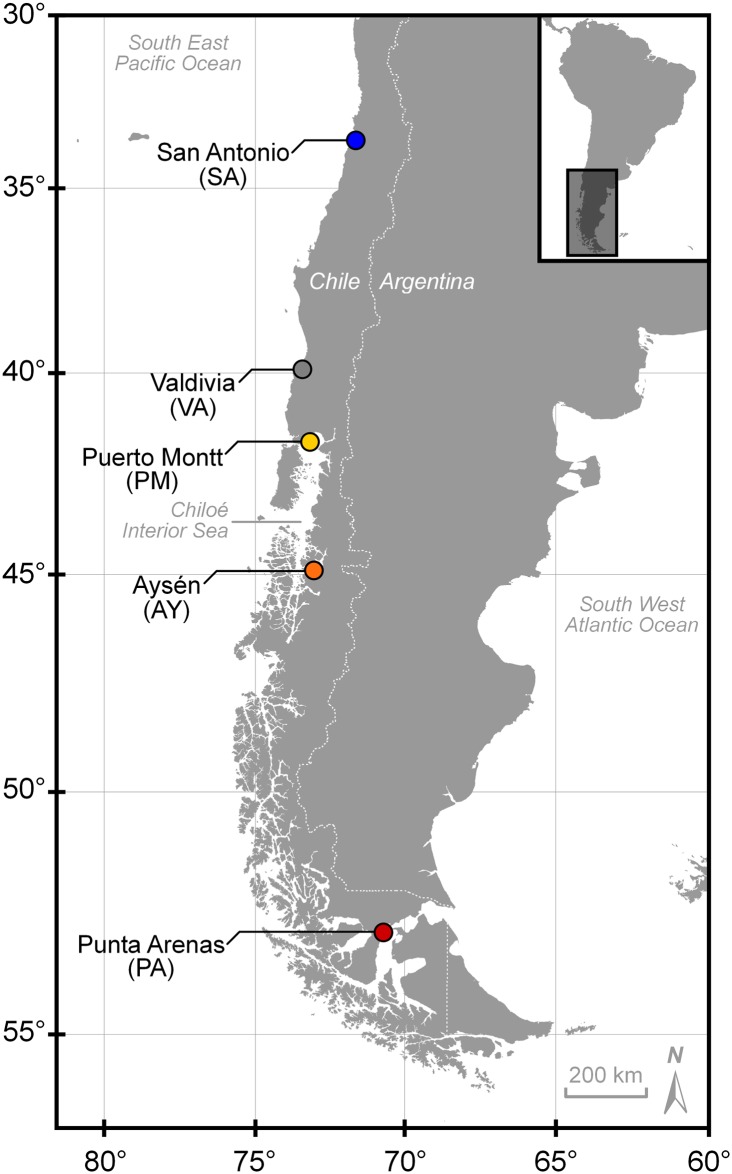
Map showing landing locations of *Zearaja chilensis* and *Dipturus trachyderma* surveyed in this study. San Antonio (blue) and Valdivia (grey) represent off-shore fishing grounds. Puerto Montt (yellow) and Aysén (orange) represent fishing grounds within the Chiloé Interior Sea. Punta Arenas (red) represent an admixture of fishing grounds between oceanic and fjord ecosystems.

**Fig 2:**
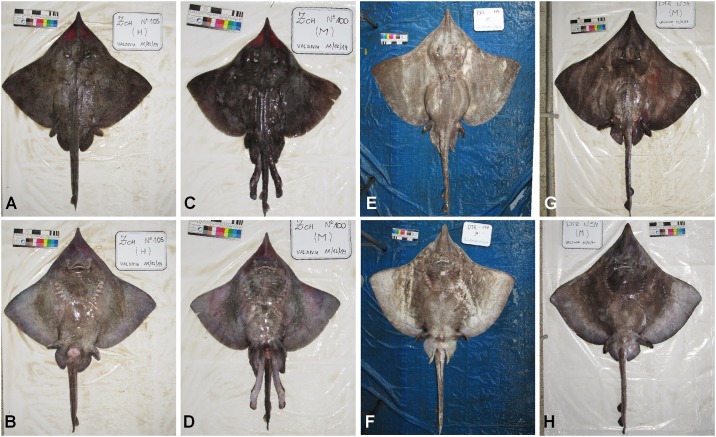
Standardized photographs used to confirm species identification of longnose skates. Dorsal and ventral views of *Zearaja chilensis* (female A, B; male C, D). Dorsal and ventral views of *Dipturus trachyderma* (female E, F; male G, H). Colorimetric scale bar = 25 cm.

*Zearaja chilensis* and *Dipturus trachyderma* were collected from five locations in the south-east Pacific Ocean, off the coast of Chile (Fig 1). The five locations represent landing sites for three separate fishing areas; San Antonio (SA, 33°35.5'S 71°37'W) and Valdivia (VA, 39°52.5'S 73°24'W) represent off-shore fishing grounds in the north; Puerto Montt (PM, 41°28.4'S 72°56.4'W) and Aysén (AY, 45°25'S 72°49'W) represent the Chiloé Interior Sea and; Punta Arenas (PA, 53°10.2'S 70°54.5'W) represents an admixture of oceanic and fjord fishing grounds in the south of Chile. Of the 408 specimens caught, 271 were *Z*. *chilensis* (101 males, 170 females) and 137 were *D*. *trachyderma* (67 males, 70 females). These specimens were used to explore the possibility of spatial genetic structure within their respective populations. This study did not involve endangered or protected species, and samples were obtained from deceased animals caught by a commercial fishery. Capture of skates was permitted under Fisheries Undersecretariat Research Permits (R. Ex.) 2878–14, 3442–14 and 1482–15, issued by the Ministry of Economy, Development, and Tourism. Landed specimens were photographed using a standardised protocol (see Fig 2) and initially identified using field guides [39, 40]. In all cases, species identification was subsequently checked using the standardised photographs. Some specimens lacked the morphological features (e.g., spinulation pattern and head shape) necessary for unambiguous identification, such as nuchal thorns or, as in juvenile males (total length, L_T_ <100 cm) [15], secondary sexual characteristics. However, DNA analysis was used to confirm species identity of all specimens.

The size (L_T_) and sex of each specimen were recorded following Last et al. [41]. A Chi-square goodness-of-fit test (χ^2^) [42] was conducted to examine whether the male to female ratio varied significantly from 1:1. Previously published size-at-maturity values were used to determine the number of mature individuals for both *Z*. *chilensis* [28] and *D*. *trachyderma* [34].

A muscle tissue sample was taken from each individual skate and stored in 100% ethanol at -20°C until required for DNA-extraction. Total genomic DNA, extracted (from ~25 mg of tissue) following a modified salting-out method [43] was used for both mitochondrial DNA (mtDNA) and microsatellite genotyping.

### Mitochondrial DNA amplification and data analysis

Mitochondrial DNA was one of two types of genetic marker used to assess genetic population structure in the two species. Novel primers were designed for the mitochondrial control region (mtCR) from the complete mitochondrial genome of *Z*. *chilensis* [44], using Primer3 in Geneious Software v.8.1 (Biomatters Ltd., Auckland). The forward primer sequence, *Zch_CR*_F (5'–TGA ACT CCC ATC CTT GGC TC –3'), was placed at *t*RNA^*Pro*^ between 15,610 bp and 15,629 bp, and the reverse primer, *Zch_CR*_R (5'–GTA TTG GTC GGT TCT CGC CA –3'), was positioned in the central control region between 16,186 base pairs (bp) and 16,205 bp. Primers were blasted against the complete mitochondrial genome and all available sequences on NCBI Genbank [45], to ensure that the sequences were unique and on target.

Initial screening of the selected primers was performed on 16 *Z*. *chilensis* individuals, and they were subsequently tested against the closely related species *D*. *trachyderma*. Approximately 600 bp of the mtCR was successfully amplified for both skate species. PCR amplifications were performed for all sampled individuals in 10 μl reactions containing 1 μl of genomic DNA (20–50 ng), 5.9 μl of Milli-Q H_2_O, 1 μl of 10x buffer MgCl_2_ (15 mM), 1 μl of dNTPs (2 mM), 0.5 μl of each primer (*Zch_CR*_F and *Zch_CR*_R), and 0.1 μl Taq DNA polymerase (5 U/μl). Thermal cycling consisted of an initial denaturation at 95°C for 1 min, followed by 35 cycles at 94°C for 1 min, 55°C for 1 min, and 72°C for 1 min. A final extension step was added at 72°C for 10 s. Amplified PCR products were treated with 1 U ExoSAP-IT (USB^®^ Products Affymetrix, Inc) at 37°C for 45 min, followed by an inactivation step at 80°C for 15 min. The cleaned PCR product was sequenced in both directions using BigDye Kit v3.1 on an ABI Prism 3130*xl* Genetic Analyser (Applied Biosystems).

Sequences were analysed using Geneious v.8.1 (Biomatters Ltd, Auckland). Each sequence was manually reviewed for uncalled and miscalled bases, and all variable positions were confirmed by comparing sequence reads produced by the forward and reverse sequences on each individual. Primer sequences were then removed, and once the sequences had been checked for discrepancies, a consensus sequence was produced for each individual. Aligned consensus sequences were used to determine the position of single nucleotide polymorphisms (SNPs) and insertion-deletion (indel) events across individuals. All sequences were deposited in GenBank under the accession numbers KX708934 to KX709341.

To characterise the mtDNA genetic diversity within and between populations, the number of haplotypes (N*h*), polymorphic sites and, haplotype (*h*) and nucleotide (π) diversity indices were calculated using DnaSP v.5.10.1 [46] for each species. Genetic differentiation among populations and pair-wise Φ_ST_ were assessed using Arlequin v3.5.1.2 [47]. The genetic distance between haplotypes was estimated using the Tamura–Nei [48] nucleotide substitution model with gamma set to 0.25. A median-joining network of haplotypes was constructed in Network v.4.6.1.3 [49] to visualise haplotype clustering and diversity.

### Microsatellite loci amplification and data analysis

To develop species-specific microsatellite genetic markers for each species, raw genomic DNA data from two voucher specimens [44, 50] were retrieved from the Genomic Database Repository, *e*Fish (*Zearaja chilensis* BioVoucher: 2014-ZCH-1004; *Dipturus trachyderma* BioVoucher: 2015-DTR-004). Sequences between 150 and 400 bp were explored for microsatellite motifs using the software QDD v.3.1 [51]. Sequences having dinucleotide and imperfect repeat motifs were excluded. The remaining sequences were considered if the number of repeat units was greater than ten. Selected sequences were blasted against the NCBI Genbank dataset to identify and exclude loci located in potential coding regions. Finally, selected forward and reverse primers were custom-blasted against the original libraries using Geneious v.8.1, to exclude primers with homology to regions outside the target flanking sequence and ensure no loci duplication. For each species, a set of 48 microsatellite loci was selected, and the 5’ end of the forward primers were tailed with a ‘CAG-tail’ to allow fluorescent labelling of PCR product while the reverse primers were tailed with ‘GTTT-tail’ to ensure complete adenylation [52].

To optimise the experimental design of population surveys with these loci, a power analysis (Powsim [53]) was used to simulate the probability of detecting population genetic differentiation across a range of expected *F*_ST_ values in two theoretical populations. Allele frequencies for 16 loci were collected in order to perform a pilot testing in the power analysis. Loci were excluded if they had (1) a major allele frequency greater than 90%, and (2) potential lower scoring accuracy due to the presence of large numbers of alleles. As recommended by Ryman & Palm [53], estimates of statistical power for a defined level of divergence was controlled by changing the generations of drift (*t*) instead of varying the effective population size (*N*_e_), which can cause the loss of low-frequency alleles. Sample sizes per population were 50, 100 and 150 and the final number of loci considered was 10. Values of *F*_ST_ were set at 0.01, 0.005 and 0.0025, which was equivalent to migration rate of 2.5%, 5% and 10%. The following parameters were used: effective population size (*N*_e_) = 1,000; number of simulations = 1,000; and generations of drift (*t*) = 20 (*F*_ST_ = 0.01), 10 (*F*_ST_ = 0.005) and 5 (*F*_ST_ = 0.0025). The degree of significant differentiation (quantified as *F*_ST_ values) for each replicate run was tested using chi-square and Fisher’s exact probability to test the null hypothesis of genetic homogeneity.

Initial screening of the selected microsatellite primers was performed on 16 individuals of each species. Samples were amplified using a PCR reaction of 11 μl containing 1 μl of genomic DNA (10–15 ng), 0.05 μl forward primer, 0.25 μl reverse primer, 0.25 μl of ‘CAG-FAM’, 5.5 μl of 2 x MyTaq mix (Bioline Australia, containing about 0.15 μl of Taq) and 3.95 μl of Milli-Q H_2_O. Thermocycler conditions were 95°C for 3 min, followed by 37 cycles of 94°C for 15 s, 57°C for 15 s and 72°C for 10 s. A final extension was performed at 72°C for 30 min and held at 15°C. PCR products were diluted 200–fold and sequenced by capillary electrophoresis on an ABI Prism 3130*xl* Genetic Analyser following the manufacturer’s recommendation. Alleles were sized against an internal size standard (GeneScan-500 LIZ) before being scored using Geneious v.8.1. Further characterisation of these loci was undertaken by genotyping 32 additional individuals of *Z*. *chilensis*; the best loci were then tested against *D*. *trachyderma* in order to have the final selection.

A total of ten microsatellite loci were found to be polymorphic and selected for genotyping the species in this study. Of these, nine were used for *Z*. *chilensis* and seven for *D*. *trachyderma*. Six out of the ten loci were common to both species in order to test for potential hybridisation, as the species overlap in latitudinal and bathymetric distribution, are morphologically similar and closely-related longnose skates of the Family Rajidae. One primer stock was set up for each locus, consisting of forward and reverse primer pairs and the corresponding fluorescently labelled CAG primer (namely Fam, Vic, Ned or Pet) and used in multiplex PCRs. Locus and colour combinations per multiplex PCR were chosen to minimise allele overlap in size. Primer stocks consisted of 6 μl of 100uM forward primer, 30 μl of 100uM reverse primer, 15 μl of 200uM of CAG-fluoro labelled primer and 99 μl Milli-Q H_2_O. For use in PCR amplifications, primer stocks were combined into a primer mix in proportions that were determined experimentally to achieve optimal amplification across all loci. Microsatellite PCR amplifications were performed in 14 μl reaction containing 2 μl of genomic DNA (10–15 ng), 4 μl of primer mix and 8 μl of 2 x MyTaq mix (Bioline, Australia containing about 0.15ul of Taq) that was added once the reaction was at 95°C in the PCR machine. Thermal conditions were the following: 95°C for 10 min, followed by 37 cycles of 94°C for 15 s, 57°C for 15 s and 72°C for 10 s. The reaction was then exposed to 72°C for 30 min and held at 12°C. Four (*Z*. *chilensis*) and three (*D*. *trachyderma*) multiplexed PCRs were performed to genotype each individual at these loci (S1 Table). The PCR products from multiple amplification reactions (one of each fluorophore) were diluted and combined in a single lane (lane-plex). Fragment separation was performed on an ABI 3130XL, and alleles were sized against an internal size standard (GeneScan-500 LIZ).

Genotypes were scored and binned using the microsatellite plug-in in Geneious software v.8.1. Micro-Checker v.2.2.3 [54] was used to check for scoring errors revealed by heterozygote deficit across loci (caused by the presence of null alleles and/or large allele dropout) and heterozygote excess (caused by incorrectly scoring stutter bands). If deviations were detected, scoring was revised until deviations were eliminated or minimised. Genepop v.4 on-the-web [55, 56] was used to assess for departures from Hardy–Weinberg equilibrium (HWE) and linkage disequilibrium (LD) among different loci; significance was accepted at *P* < 0.05. The assessment used Markov-chain parameters of 1,000 dememorisation steps, 100 batches and 1,000 iterations per batch, for multiple comparisons using Bonferroni adjustment (α = 0.05). The number of alleles (*N*_A_) and their frequencies, expected (*H*_E_) and observed heterozygosity (*H*_O_) was estimated using GenAlEx v.6.501 [57, 58].

Population pair-wise *F*_ST_s and *p*-values of the *F*_ST_ between populations were estimated using 1000 permutations in Arlequin v.3.5.2.2 [54]. Partial Mantel tests were implemented using the Isolation by Distance Web Service v.3.23 [59], with significance determined via permutation tests.

The Bayesian clustering algorithm implemented in Structure v.2.3.4 [60–63] was used to examine possible population subdivision. To determine the optimal number of cohesive groups (k) was estimated with 20 independent runs of *K* = 1–10. The length of the burn-in period was set to 100,000 iterations, followed by 100,000 Markov Chain Monte Carlo (MCMC) repetitions under the admixture model with correlated allele frequencies. The optimal number of populations was estimated using the Δ*K* method [64] implemented in Structure Harvester v.0.6.94 web-based tool [65]. Cluster Markov Packager Across *K* (CLUMPAK [66]) was used to summarise results from Structure and provide a graphical representation referring to individuals and populations through bar plots. To maximize discrimination between populations, discriminant analysis of principal components (DAPC [67]) was applied as implemented in R-package Adegenet v.1.4–2 [68, 69]. The cross-validation function *xvalDapc* was used to set the number of principal components to 50 [67]. DAPC uses a sequential *K*-means approach to determine the numbers of clusters in the data, assigning individuals to clusters independent of their collection site.

## Results

Of the 408 specimens collected, there were 11 whose identity was uncertain based on external morphology. Although initially considered to be *D*. *trachyderma* they were all identified as *Z*. *chilensis* based on their mtCR (557 bp) sequences.

The sex ratio (males: females) for *Z*. *chilensis* was significantly biased towards females (1:2.17; χ^2^ = 6.357, d.f. = 1, *P* > 0.05) in all locations except VA, where the sex ratio (1:0.906) was not significantly different to 1:1 (χ^2^ = 0.262, d.f. = 1, *P* = 0.968). Overall, body size ranged between 61 and 117 cm L_T_ for males and between 71 and 141.8 cm *L*_T_ for females. Mature females were common at PM (57%) and PA (84%) but represented only 14% of specimens from the other locations. In the case of males, approximately half of the individuals were mature (53%), with PM and PA having the highest proportions (83% and 100% respectively).

No significant differences were observed in the overall sex ratio of *D*. *trachyderma* (1:1.04; χ^2^ = 0.029, d.f. = 1, *P* = 0.965), although there was a significant bias towards females in SA (1:2.058; χ^2^ = 5.557, d.f. = 1, *P* > 0.05). However, low sample sizes for PM (14), AY (11) and PA (7) preclude a definitive interpretation of the data. Males of *D*. *trachyderma* ranged from 89 to 207.5 cm *L*_T_ and females 92.9 to 242.5 cm *L*_T_. Except for a low percentage of mature specimens in SA and VA (4.4% and 3.6% respectively), all males and females were immature.

### Mitochondrial data

#### Zearaja chilensis

A total of 271 mtCR consensus sequences of 557 bp in length were successfully obtained for *Zearaja chilensis*. Analysis of these sequences revealed 10 polymorphic sites and one 1bp indel, which together defined 11 haplotypes (Table 1). About 90% of individuals were represented by the haplotypes *Zch_CR*_1_ (45.4%) and *Zch_CR*_2_ (44.6%). With the exception of one individual found in PM, haplotype *Zch_CR*_1_ was only observed in samples from off-shore fishing grounds in the north (SA and VA) and PA in the south; and occurred in 64–72% of individuals. While haplotype *Zch_CR*_2_ was found in *Z*. *chilensis* landed at all five locations, it occurred in 96–100% of individuals from the Chiloé Interior Sea that were landed at PM and AY, in 36% of individuals from PA and only 5–13% of individuals to the north (VA and SA)(Table 1). Haplotype *Zch_CR*_3_ occurred at both northern sites but was only found in 3–4% of individuals. The remaining eight haplotypes were only found at individual sites, and in five cases was represented by a single individual.

**Table 1:** Geographic distribution of mtDNA control region haplotypes found in *Zearaja chilensi*s and *Dipturus trachyderma*.

	Position											Sampling location					
***Z*. *chilensis***		1	1	1	2	2	4	4	5	5	5	**SA**	**VA**	**PM**	**AY**	**PA**	**Total**
	9	0	2	6	0	0	1	9	1	2	2						
	9	0	1	6	1	2	9	5	2	8	9						
*Zch_CR*_1_	A	G	–	G	A	T	T	C	C	C	T	0.700	0.721	0.021	–	0.644	123
*Zch_CR*_2_	·	·	–	·	·	·	·	·	T	·	·	0.057	0.131	0.957	1.00	0.356	121
*Zch_CR*_3_	G	·	–	·	·	·	·	·	·	·	·	0.043	0.033	–	–	–	5
*Zch_CR*_4_	·	A	T	·	·	·	·	·	·	T	C	0.100	**–**	–	–	–	7
*Zch_CR*_5_	·	·	–	·	G	·	·	·	T	·	·	**–**	0.082	–	–	–	5
*Zch_CR*_6_	·	·	–	A	G	·	·	·	T	·	·	0.071	**–**	–	–	–	5
*Zch_CR*_7_	·	·	–	·	·	C	·	·	·	·	·	0.014	**–**	–	–	–	1
*Zch_CR*_8_	·	·	–	·	·	·	C	·	T	·	·	**–**	0.016	–	–	–	1
*Zch_CR*_9_	·	·	–	A	·	·	·	·	·	·	·	0.014	**–**	–	–	–	1
*Zch_CR*_10_	·	·	–	·	·	·	·	T	T	·	·	**–**	**–**	0.021	–	–	1
*Zch_CR*_11_	·	·	–	·	·	·	C	·	·	·	·	**–**	0.016	–	–	–	1
***D*. *trachyderma***											*N* =	70	61	47	48	45	271
	1	1	1	2	2	2	3	4	5			**SA**	**VA**	**PM**	**AY**	**PA**	**Total**
	4	7	7	0	4	7	0	0	4								
	9	6	8	1	5	1	5	2	9								
*Dtr_CR*_1_	T	T	T	G	A	T	G	T	T			0.981	0.811	0.857	1.00	1.00	124
*Dtr_CR*_2_	C	C	C	A	T	A	A	·	G			**–**	0.132	**–**	**–**	**–**	7
*Dtr_CR*_3_	C	·	·	·	·	·	·	C	·			0.019	0.038	0.071	**–**	**–**	4
*Dtr_CR*_4_	C	·	·	·	·	·	·	·	·			**–**	0.019	0.071	**–**	**–**	2
											*N* =	52	53	14	11	7	137

Samples per locality and haplotypes are indicated as the proportion of the total number of specimens with that haplotype. Sampling localities: SA, San Antonio; VA, Valdivia; PM, Puerto Montt; AY Aysén; and PA, Punta Arenas.

To test whether mtDNA genetic diversity was homogeneous across populations, off-shore (SA and VA) and PA populations were compared to those in the Chiloé Interior Sea (PM and AY) (Table 2). Haplotype diversity was similar across off-shore locations and PA (*h* = 0.46–0.50), but was significantly different to those in the interior sea: PM (*h* = 0.08) and AY (*h* = 0.00). While nucleotide diversity was low for all locations, it was extremely low in the Chiloé Interior Sea compared to off-shore fishing grounds and PA.

**Table 2:** Genetic diversity per sampling locality from mtDNA control region and microsatellite loci for *Zearaja chilensis* and *Dipturus trachyderma*.

	mtDNA haplotypes				Microsatellites				
*Z*. *chilensis*	*N*	*N*_h_	*H*	π	*N*	*N*_A_	*H*_O_	*H*_E_	*F*_IS_
SA	70	7	0.496 ± 0.069	0.0024 ± 0.0017	30	10	0.603	0.613	0.026
VA	61	6	0.462 ± 0.073	0.0012 ± 0.0010	32	9	0.576	0.593	0.021
PM	47	3	0.084 ± 0.055	0.0001 ± 0.0003	32	10	0.578	0.576	0.002
AY	48	1	0.000	0.000	32	7	0.534	0.546	0.004
PA	45	2	0.469 ± 0.044	0.0008 ± 0.0008	28	8	0.602	0.599	-0.023
***D*. *trachyderma***									
SA	52	2	0.038 ± 0.037	0.0001 ± 0.0003	32	5	0.421	0.432	0.049
VA	53	4	0.329 ± 0.077	0.0036 ± 0.0023	31	6	0.360	0.376	0.022
PM	14	3	0.275 ± 0.148	0.0007 ± 0.0008	14	3	0.471	0.440	-0.033
AY	11	1	0.000	0.000	11	3	0.414	0.390	0.015
PA	7	1	0.000	0.000	7	3	0.347	0.322	-0.096

Number of individuals (*N*); number of mtCR haplotypes (*N*_h_); haplotype diversity ± standard deviation (*h*); nucleotide diversity ± S.D. (π); maximum number of alleles observed per locus (*N*_A_); observed (*H*_O_) and expected heterozygosity (*H*_E_); Wright’s inbreeding coefficient of individuals relative to the subpopulation (*F*_IS_). Values for microsatellite data are presented as the average across loci. Sampling locations as in Table 1.

**Fig 3:**
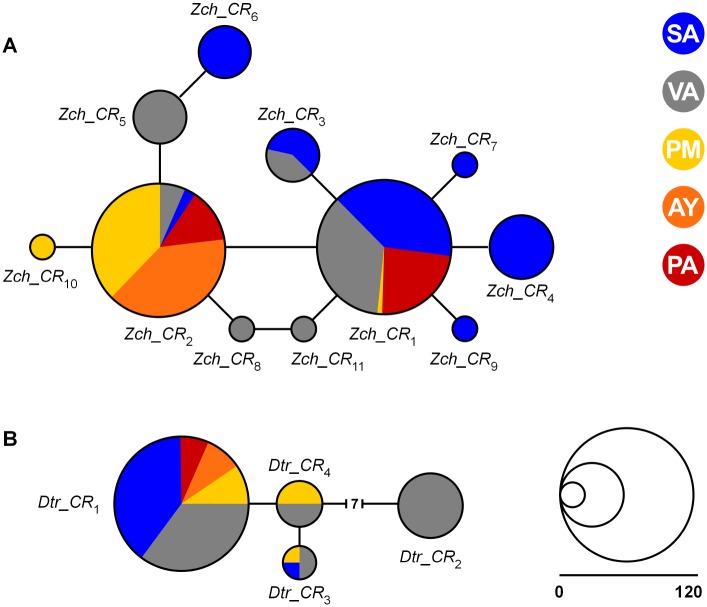
Median-joining network of mtCR haplotypes for (A) *Zearaja chilensis* and (B) *Dipturus trachyderma*. Haplotypes are represented by circles with size proportional to frequency in the total sample. All branches correspond to one mutation except where indicated otherwise. Sampling locations: San Antonio (SA, blue), Valdivia (VA, grey), Puerto Montt (PM, yellow), Aysén (AY, orange) and Punta Arenas (PA, red).

The median-joining network for *Z*. *chilensis* subdivided the mtCR haplotypes into two main groups: one composed of haplotypes mostly from PM and AY and the other of haplotypes mostly from SA, VA, and PA (Fig 3A). Each group was the centre of a star-like cluster with more haplotypes diverging from them by two (or more) mutations. Comparisons of *Φ*_ST_ between locations suggest a significant genetic differentiation between the Chiloé Interior Sea (PM and AY), and off-shore locations (SA and VA) and PA (*P* < 0.05; Table 3).

**Table 3:** Estimates of pair-wise genetic distance (*F*_ST_) (below diagonal) among locations for mtDNA control region (mtCR) sequences and microsatellites loci for *Zearaja chilensis* and *Dipturus trachyderma*. *F*_ST_ *P*-values are indicated above diagonal.

Species		Location				
		SA	VA	PM	AY	PA
***Z*. *chilensis***						
mtCR						
	**SA**	–	0.009	0.000	0.000	0.000
	**VA**	**0.036**	–	0.000	0.000	0.126
	**PM**	**0.500**	**0.583**	–	0.756	0.000
	**AY**	**0.525**	**0.623**	0.008	–	0.000
	**PA**	**0.079**	0.023	**0.584**	**0.644**	–
Microsatellite loci						
	**SA**	–	0.666	0.061	0.105	0.030
	**VA**	-0.002	–	0.406	0.016	0.003
	**PM**	0.008	0.000	–	0.723	0.076
	**AY**	0.007	**0.012**	-0.003	–	0.103
	**PA**	**0.011**	**0.019**	0.007	0.006	–
***D*. *trachyderma***						
mtCR						
	**SA**	–	0.000	0.126	0.990	0.990
	**VA**	**0.110**	–	0.369	0.198	0.432
	**PM**	0.074	0.027	–	0.513	0.765
	**AY**	-0.045	0.037	0.029	–	0.990
	**PA**	-0.075	0.006	-0.015	0.000	–
Microsatellite loci						
	**SA**	–	0.131	0.358	0.996	0.166
	**VA**	0.011	–	0.028	0.608	0.436
	**PM**	0.002	**0.030**	–	0.248	0.072
	**AY**	-0.035	-0.007	0.010	–	0.607
	**PA**	0.020	0.0001	0.041	-0.017	–

Significant *Φ*_ST_ and *F*_ST_ values are presented in bold. Significance accepted at *P* < 0.05. Sampling locations are described in Table 1.

#### Dipturus trachyderma

Sequence data (556 bp of the mtCR) was obtained for 137 *D*. *trachyderma*. Among individuals, a total of nine polymorphic sites were detected, representing four haplotypes. All differences were due to substitutions and no indels were found (Table 1). Haplotype frequencies clearly showed that *Dtr_CR*_1_ was the dominant haplotype, found in 91% of individuals. This haplotype occurred at all collection sites and at high frequency; 81–100% of specimens within any given region. VA was the only location where all four haplotypes were observed, and haplotype *Dtr_CR*_2_ was only detected at this location. Haplotypes *Dtr_CR*_3_ and *Dtr_CR*_4_ occurred at frequencies of 7% or less and were not present at AY or PA. Differences in frequencies may be related to the restricted sampling size of PM, AY, and PA.

The mean haplotype and nucleotide diversities for specimens from the three most northern sites followed a similar pattern: VA > PM >> SA, ranked from highest to lowest diversity (Table 2). AY and PA were excluded from this analysis as only a single haplotype was present in these locations.

The median-joining network of mtCR haplotypes for *D*. *trachyderma* shows a general lack of population structure (Fig 3B). Four distinct haplotypes were detected with *Dtr_CR*_1_ being the most prevalent haplotype present in all locations. Only one mutation was observed between all haplotypes, with exception of *Dtr_CR*_2._ This haplotype was only found in 13% of individuals, exclusively found in VA and separated by seven mutations from all remaining haplotypes. Comparisons of pair-wise *Φ*_ST_ values revealed a low and non-significant pair-wise divergence among locations (Table 3).

### Microsatellite data

#### Zearaja chilensis

A total of 154 individuals were genotyped with 9 microsatellite loci in the nuclear DNA (nDNA). Tests for scoring error indicated the possibility of null alleles for only one locus (*Zch_MS*_10_) at a single location (AY) due to an excess of homozygotes. Overall, no loci showed significant departure from HWE or evidence for LD. However, three loci deviated from HWE (*P* < 0.05); *Zch_MS*_16_ in VA, *Zch_MS*_13_ in PM and *Zch_MS*_19_ in PA. The exclusion of these loci at these sites did not change the pattern of differentiation between populations and therefore these loci were included in the study.

Across all sampled populations, the allelic diversity ranged from two (*Zch_MS*_13_) to 10 alleles (*Zch_MS*_31_) (Table 4). Overall, expected (*H*_E_) and observed heterozygosity (*H*_O_) values ranged between 0.300 and 0.810. The highest *H*_O_ was registered for locus *Zch_MS*_19_ whereas locus *Zch_MS*_13_ showed the lowest *H*_O_ and *H*_E_ values (Table 4). A number of alleles, *H*_O_ and *H*_E_ varied among populations at different loci. Observed number of alleles within locations ranged between two in SA at locus *Zch_MS*_13_ and, 10 in SA and PM at locus *Zch_MS*_31_ (Table 5). The highest mean of *H*_O_ and *H*_E_ was observed in SA (0.603 and 0.613 respectively) and the lowest in AY (*H*_O_ 0.534; *H*_E_ 0.546).

**Table 4:** Characterisation of polymorphic microsatellite loci used for *Zearaja chilensis* (n = 9) and *Dipturus trachyderma* (n = 7) in this study.

Locus	GenBank accession number	Repeat Motif	Size (bp)	*Z*. *chilensis*			*D*. *trachyderma*		
				*N*_A_	*H*_E_	*H*_O_	*N*_A_	*H*_E_	*H*_O_
*Zch_MS*_06_	KX708924	(ATC)^8^	137	8	0.452	0.474	2	0.253	0.289
*Zch_MS*_08_	KX708925	(AC)^10^	143	5	0.540	0.545	4	0.387	0.445
*Zch_MS*_10_	KX708926	(AGC)^7^	333	4	0.650	0.614	—	—	—
*Zch_MS*_13_	KX708927	(AC)^7^	166	5	0.302	0.300	—	—	—
*Zch_MS*_15_	KX708928	(AATG)^7^	141	5	0.609	0.599	2	0.475	0.463
*Zch_MS*_16_	KX708929	(AC)^10^	301	7	0.622	0.578	4	0.209	0.142
*Zch_MS*_19_	KX708930	(AAT)^8^	213	7	0.797	0.810	4	0.419	0.475
*Zch_MS*_29_	KX708931	(AC)^9^	394	7	0.496	0.518	—	—	—
*Zch_MS*_31_	KX708932	(AC)^7^	182	10	0.800	0.768	6	0.568	0.580
*Dtr_MS*_08_	KX708933	(ACTC)^13^	329	—	—	—	6	0.468	0.434

Locus name, primer sequences, repeat motif, allele size, maximum number of alleles (*N*_A_) and expected (*H*_E_) and observed (*H*_O_) heterozygosities. (—) Indicates microsatellite loci not used on the species.

**Table 5:** Genetic diversity per locality and microsatellite locus for *Zearaja chilensis* and *Dipturus trachyderma*.

Locus	*Z*. *chilensis*						*D*. *trachyderma*				
		SA	VA	PM	AY	PA	SA	VA	PM	AY	PA
*Zch_MS*_06_	***N***_**A**_	7	6	5	5	5	2	2	2	2	2
	***H***_**E**_	0.550	0.515	0.451	0.353	0.464	0.285	0.144	0.293	0.298	0.245
	***H***_**O**_	0.467	0.594	0.469	0.375	0.393	0.281	0.156	0.357	0.364	0.286
*Zch_MS*_08_	***N***_**A**_	5	5	5	5	4	2	4	2	2	2
	***H***_**E**_	0.528	0.437	0.490	0.593	0.650	0.469	0.389	0.459	0.636	0.133
	***H***_**O**_	0.500	0.438	0.484	0.625	0.679	0.438	0.438	0.571	0.483	0.143
*Zch_MS*_10_	***N***_**A**_	4	4	4	4	4	—	—	—	—	—
	***H***_**E**_	0.668	0.680	0.656	0.627	0.641	—	—	—	—	—
	***H***_**O**_	0.577	0.688	0.636	0.438	0.714	—	—	—	—	—
*Zch_MS*_13_	***N***_**A**_	2	3	4	4	3	—	—	—	—	—
	***H***_**E**_	0.262	0.225	0.314	0.301	0.406	—	—	—	—	—
	***H***_**O**_	0.241	0.219	0.233	0.344	0.464	—	—	—	—	—
*Zch_MS*_15_	***N***_**A**_	4	5	4	5	4	2	2	2	2	2
	***H***_**E**_	0.587	0.602	0.639	0.626	0.571	0.458	0.451	0.497	0.469	0.500
	***H***_**O**_	0.633	0.563	0.677	0.548	0.589	0.516	0.313	0.308	0.650	0.429
*Zch_MS*_16_	***N***_**A**_	4	5	6	4	5	2	4	2	2	2
	***H***_**E**_	0.665	0.669	0.645	0.517	0.536	0.242	0.272	0.293	0.236	0.139
	***H***_**O**_	0.600	0.563	0.710	0.548	0.551	0.156	0.250	0.214	0.091	0.147
*Zch_MS*_19_	***N***_**A**_	6	7	6	6	6	3	4	3	3	2
	***H***_**E**_	0.787	0.797	0.797	0.811	0.667	0.467	0.484	0.589	0.310	0.245
	***H***_**O**_	0.852	0.750	0.875	0.906	0.796	0.438	0.469	0.798	0.182	0.286
*Zch_MS*_29_	***N***_**A**_	6	6	3	5	5	—	—	—	—	—
	***H***_**E**_	0.647	0.625	0.355	0.387	0.541	—	—	—	—	—
	***H***_**O**_	0.690	0.625	0.306	0.359	0.536	—	—	—	—	—
*Zch_MS*_31_	***N***_**A**_	10	9	10	7	8	4	6	3	3	3
	***H***_**E**_	0.818	0.788	0.842	0.726	0.786	0.572	0.584	0.571	0.539	0.612
	***H***_**O**_	0.867	0.750	0.806	0.633	0.828	0.567	0.567	0.533	0.625	0.571
*Dtr_MS*_08_	***N***_**A**_	—	—	—	—	—	5	5	3	3	3
	***H***_**E**_	—	—	—	—	—	0.533	0.471	0.417	0.398	0.520
	***H***_**O**_	—	—	—	—	—	0.552	0.379	0.273	0.250	0.714

Sampling localities are as in Table 1.

**Fig 4:**
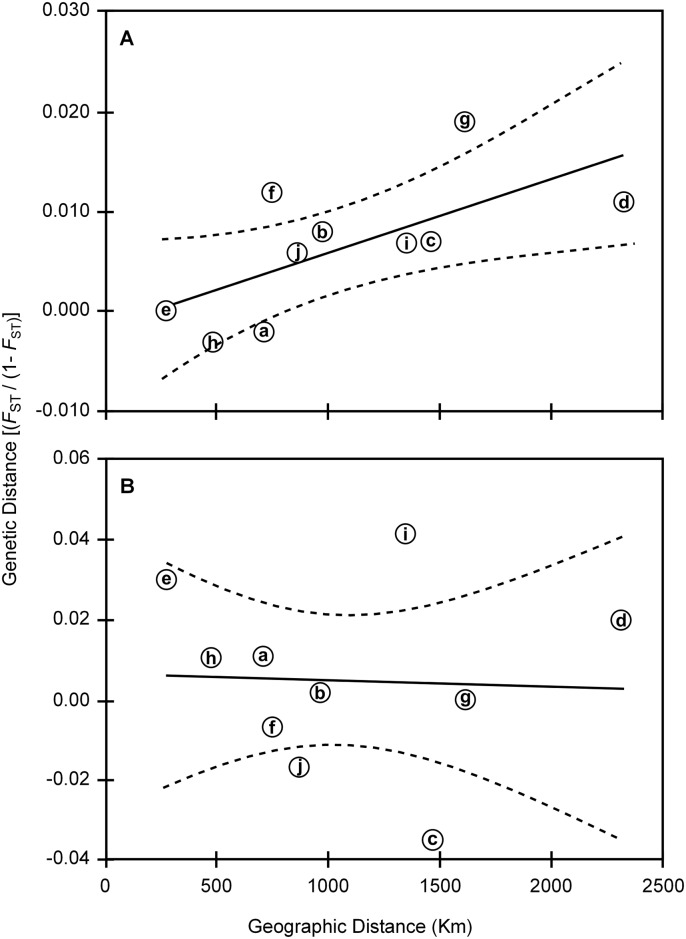
Correlation between genetic and geographic distance, for populations of (A) *Zearaja chilensis* and (B) *Dipturus trachyderma*. Linear adjustment (solid line) and 95% confidence interval (dashed line) are specified. Geographic distance are indicated by paired locations, (a) SA and VA; (b) SA and PM; (c) SA and AY; (d) SA and PA; (e) VA and PM; (f) VA and AY; (g) VA and PA; (h) PM and AY; (i) PM and PA; (j) AY and PA. Sampling locations abbreviations as in Fig 3.

**Fig 5:**
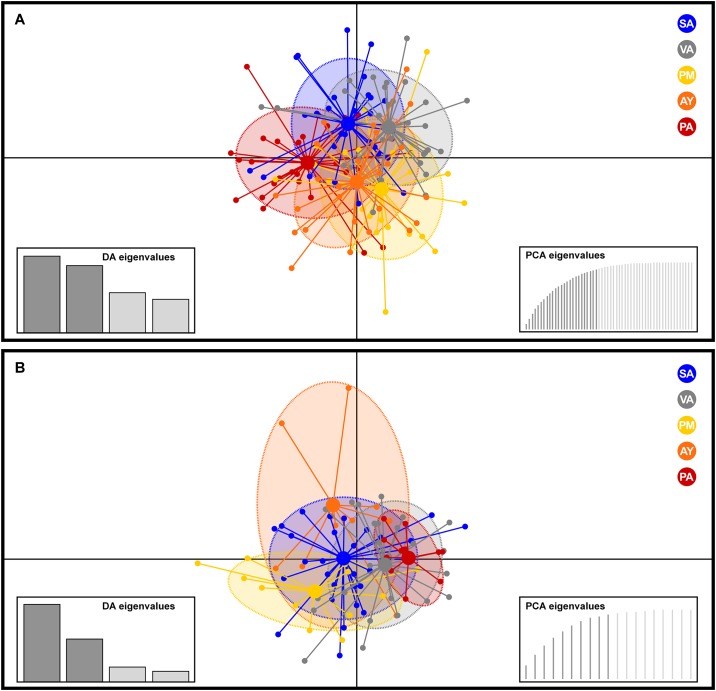
Scatterplots of the Discriminant Analysis of Principal Components (DAPC) from microsatellite genotypes for populations of (A) *Zearaja chilensis* and (B) *Dipturus trachyderma*. Dots represent individuals, whereas coloured ellipses correspond to geographical populations. Sampling locations: San Antonio (SA, blue), Valdivia (VA, grey), Puerto Montt (PM, yellow), Aysén (AY, orange) and Punta Arenas (PA, red).

The power analysis for detecting genetic differentiation using ten microsatellite loci indicated that a pair-wise *F*_ST_ of 0.01 could be detected 89% of the time at a sample size of 50 or less; for smaller *F*_ST_s of 0.005 and 0.0025, the percentage fell to 52% and 21% respectively. However, the probability of detecting population subdivision could be increased at a higher sampling size. For example, with a sample size of 100 individual and *F*_ST_-values as low as 0.0025 could be detected 77% of the time. Global *F*_ST_ is low and non-significant (*F*_ST_ 0.00907, *P*-value = 0.106). Pairwise *F*_ST_ tests indicated low, non-significant genetic differentiation among *Z*. *chilensis* sampling locations (-0.003 and 0.019) (Table 3). However, significant genetic differentiation was found between the off-shore locations (SA and VA) and PA, as well as between VA and AY. The correlation between genetic [*F*_ST_ (1—*F*_ST_)^-1^] and geographic distance was also significant (*P* < 0.05, r^2^ = 0.049), supporting the hypothesis of isolation by distance of gene flow (Fig 4A). Population assignment analyses using Structure were inconclusive as no signal of genetic differentiation between collection locations was observed (results not shown). One main cluster was identified using DAPC (Fig 5A) and, a subdivision within this cluster was observed. Individuals from off-shore locations (SA and VA) were most closely co-located; the Chiloé Interior Sea (PM and AY) centroids were also close, and the PA centroid sat off to one side. These relationships reflect the outcomes of the mtDNA analyses, but similarities should be interpreted cautiously as the microsatellite data ellipses have considerable overlap.

#### Dipturus trachyderma

Microsatellites alleles were analysed for 10 microsatellite loci for 95 individuals. Initial scoring identified three loci, *Zch_MS*_13_, *Zch_MS*_10_ and *Zch_MS*_29_ as monomorphic in this species and was thus excluded from further analysis. Evidence of scoring error was detected due to an excess of homozygotes at two loci (*Zch_MS*_16_ in SA and *Zch_MS*_15_ in VA). Deviation from HWE (*P* < 0.05) was observed at these loci and at locus *Zch_MS*_19_ in PM. However, no evidence of linkage disequilibrium was detected at any loci.

In general, allelic diversity in *D*. *trachyderma* was lower than in *Z*. *chilensis* for the same loci ranging from two (*Zch_MS*_6_ and *Zch_MS*_15_) to six alleles (*Zch_MS*_31_ and *Dtr_MS*_8_) across all sampled locations (Table 4). Overall, *H*_E_ and *H*_O_ values ranged between 0.142 and 0.580. However, the highest and lowest *H*_O_ and *H*_E_ values were registered for locus *Zch_MS*_31_ and *Zch_MS*_16_ respectively (Table 4). Among sampling locations the observed number of alleles, *H*_O_ and *H*_E_ varied at different loci (Table 5). SA and VA were the most variable in terms of allele number, however most locations had an average of two alleles.

The POWSIM analysis indicated that a pair-wise *F*_ST_ of 0.01 could be detected in 85% whereas a lower *F*_ST_ of 0.0025 could only be detected 19% of the time. Overall, global *F*_ST_ is low and non-significant (*F*_ST_ 0.00951, *P*-value = 0.143) and pairwise *F*_ST_ test indicated no significant genetic differentiation among sampling locations (-0.035 to 0.041). However, significant differences were found between VA and PM (Table 3). Comparisons of pairwise *F*_ST_ values revealed no significant isolation by distance (IBD) (*P* = 0.651, r^2^ = 0.040) (Fig 4B). DAPC analysis grouped all locations into a single cluster in agreement with previous results supporting the lack of population structure of *D*. *trachyderma* in Chilean waters (Fig 5B).

## Discussion

This study provides a genetic analysis of populations of *Zearaja chilensis* and *Dipturus trachyderma*, two economically important elasmobranch fishes in South America. Mitochondrial and nuclear markers were used to provide insight into connectivity, genetic diversity and population structure along the Chilean coast from San Antonio in the north to Punta Arenas in the south.

The microsatellite loci (nDNA) analysis was not informative enough as expected for a nuclear marker to assess the stock structure of longnose skates. However, it is interesting that mitochondrial genome (mtDNA) provides resolution on population structure, even though mtDNA variation in elasmobranchs is notoriously low [70] and relatively low numbers of SNPs were found in the mtCR of these two longnose skates.

Our data revealed that populations of *Z*. *chilensis* might comprise three potential management units, whereas a single-unit harvest management strategy is currently employed in this fishery. For *Z*. *chilensis*, there were significant differences among all locations with the exception of Puerto Montt and Aysén. The latter two are located in the Chiloé Interior Sea and hence, might be expected to be a single, relatively isolated population. The pattern of mtDNA haplotype diversity among localities emphasises the likely susceptibility of longnose skates to local overexploitation. At Aysén, only mtCR haplotype *Zch_CR*_2_ was found in 100% of the individuals and at Puerto Montt it was found in 96%, emphasising the potential isolation of the Chiloé Interior Sea due to oceanographic features, such as cold subsurface currents, high freshwater contribution from precipitation and river discharges, and high depth profile (50 to 400 m) [71]. Given the maternal inheritance of mtDNA these data indicate that there is little or no movement of males and females of *Z*. *chilensis* into the Chiloé Interior Sea and, as such, population resilience will rely almost exclusively on reproduction and self-recruiting by within- Chiloé Interior Sea adults.

Fuentealba & Leible [72], reported that longnose skates in the Arauco Gulf (between San Antonio and Valdivia) represented a major fishing resource for the region between 1980 and 1990. However, this fishery is currently non-existent, with an apparent local depletion due to a lack of management leading to the loss of large, breeding females [72]. This outcome provides a cautionary lesson whereby local population recovery is highly dependent on the species’ intrinsic resilience rather than migration from adjacent populations or stocks. In the case of longnose skates, this resilience is considered to be low leading to slow recovery from over-exploitation. The longnose skates population inhabiting Chiloé Interior Sea may be on the brink of collapse if current exploitation levels are maintained.

On the other hand, there were fewer mtDNA breaks in gene flow for *D*. *trachyderma*. The main disruption is detected between San Antonio and Valdivia in the north of the sampling area. Interestingly, 13% of specimens from Valdivia possessed a highly divergent haplotype. However, no significant population structure was found with mtDNA or microsatellite markers. Unfortunately, the low sample sizes for the Chiloé Interior Sea and Punta Arenas impeded resolution of population structure, and further research is required to assess the dispersal and connectivity of *D*. *trachyderma* along the Chilean coast.

The finding of genetic breaks between populations with mtDNA, but not with microsatellites for *Z*. *chilensis* may suggest a lack of power in the microsatellite analysis. The power analysis conducted for this species shows that *F*_ST_ down to 0.01 could be detected with the sample sizes used here (n = 50 or less). Indeed, three population pairs were separated by significant *F*_ST_ of this magnitude, but *F*_ST_ lower than 0.01 measured in this study were not significant.

Aspects of the biology of chondrichthyan fishes may have the potential to influence the genetic findings such as the possibility of female regional philopatry to inshore egg-laying grounds. The mtDNA subdivision among locations may have been the result of such movements, with our samples comprising mostly juveniles that result directly from this reproductive behaviour. Natal philopatry is well-recognised among various marine vertebrates including elasmobranch fishes, but while it has been shown definitively for sharks [16, 73–76], the situation in skates is less clear. Tagging studies have indicated that skates may have the potential to travel hundreds of kilometres [77, 78], however, 90% of individuals had a relatively small home range (within a 100 km^2^ area) [79–81]. These observations are consistent with the regional population structure observed for *Z*. *chilensis*, although we cannot confirm whether these migrations reflect small-scale movements due to preference and selection of particular habitats (e.g. nursery sites) or to natal philopatry.

Longnose skates are oviparous, producing egg capsules that contain a single embryo which spends weeks in the environment before hatching [82, 83]. Also, females are able to store sperm, and mating may occur months before the return to proposed egg-laying or nursery grounds [84]. The egg capsules of *Z*. *chilensis* and *D*. *trachyderma* are among the largest in marine fishes [85, 86] and, probably as a result of this, fecundity is low [15, 28]. The morphology of the egg capsules of both species show adaptations that suggest they are laid on soft substrates, such as coastal sediments or lodged in rocky inshore reefs [85, 86]. Given these biological traits and our results, it is highly probable that mature females do not inhabit in the fishing grounds surveyed along the Chilean coast all year round but move between inshore nursery and offshore “adult” habitats. Mature female longnose skates appear to be transient visitants of coastal areas to lay their egg capsules and it is during these inshore migrations they are particularly vulnerable to fishing activity.

The genotypes from microsatellite loci were not particularly informative regarding the spatial extent of stocks. We believe this is due to mating during the dispersal stage (non-egg-laying), where both sexes move into deeper water away from the coast as well as alongshore to seek suitable feeding habitats. Adult *D*. *trachyderma* inhabit deeper waters than *Z*. *chilensis* [32] and have been rarely sampled in the area, i.e., the egg capsule of *D*. *trachyderma* was described from the only mature female observed in Valdivia over a 10-year period of sampling (CB, *Pers*. *Obs*.). The smaller skate species (*Z*. *chilensis*) may have lower dispersal, with corresponding smaller home ranges and without extensive migrations; in contrast, the much larger *D*. *trachyderma* might be expected to have a greater dispersal potential. This proposed size of the home ranges is correlated with the pronounced genetic isolation by distance signature for *Z*. *chilensis* inferred from the microsatellite data. This relationship occurs when random mating does not occur within the entire population. Instead, there is an increased likelihood of mating among individuals in a local region as a consequence of the constrained dispersal potential of the species.

**Fig 6:**
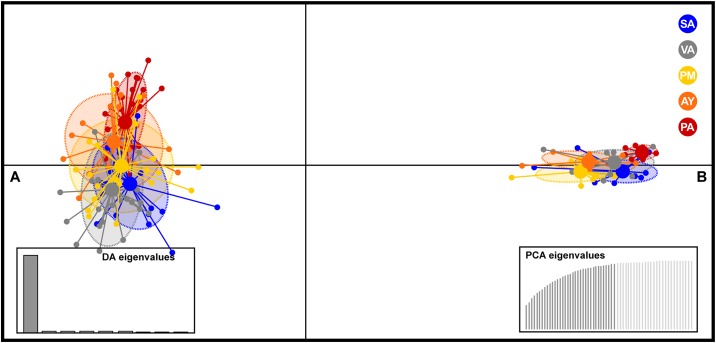
Scatterplots of the Discriminant Analysis of Principal Components (DAPC) from microsatellites genotypes comparing (A) *Zearaja chilensis* and (B) *Dipturus trachyderma*. Dots represent individuals, whereas coloured ellipses correspond to geographical populations. Sampling locations: San Antonio (SA, blue), Valdivia (VA, grey), Puerto Montt (PM, yellow), Aysén (AY, orange) and Punta Arenas (PA, red).

The set of microsatellite loci used was carefully designed to detect any hybrids between the species, and discard this plausible hypothesis (hybridisation) as individuals of cryptic morphotypes have been reported for *Z*. *chilensis* in the past [87]. The use of the same set of microsatellite loci in both species has confirmed the distinctiveness of the species identity (Fig 6) and provides an alternative to mtDNA sequencing for accurate species identification of, for example, unidentified ‘skate wings’. The separate species status has also been confirmed by phylogenomic reconstruction using whole mitochondrial genomes for both species [44, 50].

The synergy between genetic results and our understanding of biological features of longnose skates significantly increases the validity of our findings. For example, the addition of new genetic data has confirmed previous inferences about the reproductive and dispersal biology of longnose skates. The population dynamics uncovered represent an initial step towards an integrated stock and potential population connectivity assessment between South American countries. Our results elucidate new information for the sustainable management of the Chilean skate resources; however, appropriate management and enforcement actions from the Chilean government are needed to safeguard the species.

Our results provide evidence for three management units (San Antonio-Valdivia, Chiloé Interior Sea, and Punta Arenas), and the spatial stock status for *Z*. *chilensis* should be considered constrained and separate management arrangements are required for each of the management units detected. Currently, both longnose skates are caught by one single fishery widespread along the Chilean coast but only management measurements are enforced for *Zearaja chilensis*. There are no grounds to discriminate the extant population of *Dipturus trachyderma* as separate management units. However, as the fishery cannot positively separate any of the target species [13], we suggested that the same management units proposed for *Z*. *chilensis* to be considered for *D*. *trachyderma* following the precautionary principle. The actual fishery management strategy and the low genetic diversity for *D*. *trachyderma*, raise concern over the conservation status of this species. The lack of genetic evidence for population subdivision appears to correspond with their higher dispersal ability and more offshore habitat preference.

## Supporting information

S1 Table Amount of primer stock per microsatellite locus added to primer mix to set up multiplexed PCRs for genotyping *Z*. *chilensis* and *D*. *trachyderma*. Multiplex numbers are indicated in brackets.
(DOCX)
