# Supplementary material for: Towards sustainable fishery management for skates in South America: The genetic population structure of Zearaja chilensis and Dipturus trachyderma (Chondrichthyes, Rajiformes) in the south-east Pacific Ocean
Source: PLoS One. 2017 Feb 16;12(2):e0172255. doi: 10.1371/journal.pone.0172255 (PMC5313215; doi:10.1371/journal.pone.0172255)
Supplement: S1 Table — Multiplex numbers are indicated in brackets. (DOCX) [file pone.0172255.s001.docx]

**Table S1.** Amount of primer stock per microsatellite locus added to primer mix to set up multiplexed PCRs for genotyping *Z. chilensis* and *D. trachyderma*. Multiplex numbers are indicated in brackets.

| **Multiplexed PCR ID** | **Locus Number (Lab Code)** | **Locus name** | **Primer stock per single reaction (μL)** |
| --- | --- | --- | --- |
| ***Zearaja chilensis*** | | | |
| CAG-VIC (M_1) | *Zch_MS*_08_ | *Zch*_067 | 0.5 |
| CAG-VIC (M_1) | *Zch_MS*_13_ | *Zch*_070 | 0.5 |
| CAG-VIC (M_1) | *Zch_MS*_19_ | *Zch*_019 | 0.8 |
| CAG-NED (M_2) | *Zch_MS*_15_ | *Zch*_059 | 0.2 |
| CAG-NED (M_2) | *Zch_MS*_31_ | *Zch*_085 | 0.5 |
| CAG-PET (M_3) | *Zch_MS*_06_ | *Zch*_007 | 0.5 |
| CAG-PET (M_3) | *Zch_MS*_16_ | *Zch*_072 | 1.2 |
| CAG-FAM (M_4) | *Zch_MS*_10_ | *Zch*_057 | 0.9 |
| CAG-FAM (M_4) | *Zch_MS*_29_ | *Zch*_083 | 1 |
| ***Dipturus trachyderma*** | | | |
| CAG-VIC (M_1) | *Zch_MS*_08_ | *Zch*_067 | 0.6 |
| CAG-VIC (M_1) | *Zch_MS*_19_ | *Zch*_019 | 0.9 |
| CAG-NED (M_2) | *Zch_MS*_15_ | *Zch*_059 | 0.2 |
| CAG-NED (M_2) | *Zch_MS*_31_ | *Zch*_085 | 0.5 |
| CAG-NED (M_2) | *Dtr_MS*_08_ | *Dtr*_009 | 0.8 |
| CAG-PET (M_3) | *Zch_MS*_06_ | *Zch*_007 | 0.5 |
| CAG-PET (M_3) | *Zch_MS*_16_ | *Zch*_072 | 1.2 |
